# Evolutionary rescue model informs strategies for driving cancer cell populations to extinction

**DOI:** 10.1371/journal.pcbi.1014803

**Published:** 2026-09-18

**Authors:** Amjad Dabi, Joel S. Brown, Robert A. Gatenby, Corbin D. Jones, Daniel R. Schrider

**Affiliations:** 1 Department of Genetics, University of North Carolina, Chapel Hill, North Carolina, United States of America; 2 Department of Cancer Biology and Evolution, Moffitt Cancer Center, Tampa, Florida, United States of America; 3 Department of Integrated Mathematical Oncology, Moffitt Cancer Center, Tampa, Florida, United States of America; 4 Diagnostic Imaging Department, Moffitt Cancer Center, Tampa, Florida, United States of America; 5 Department of Biology, University of North Carolina, Chapel Hill, North Carolina, United States of America; 6 Integrative Program for Biological and Genome Sciences, University of North Carolina, Chapel Hill, North Carolina, United States of America; 7 UNC Lineberger Comprehensive Cancer Center, University of North Carolina, Chapel Hill, North Carolina, United States of America; Universitat zu Koln, GERMANY

## Abstract

Cancers exhibit a remarkable ability to develop resistance to a range of treatments, often resulting in relapse following first-line therapies and significantly worse outcomes for subsequent treatments. While our understanding of the mechanisms and dynamics of the emergence of resistance during cancer therapy continues to advance, questions remain about how to minimize the probability that resistance will evolve, thereby improving long-term patient outcomes. Here, we present an evolutionary simulation model of a clonal population of cells that can acquire resistance mutations to one or more treatments. We leverage this model to examine the efficacy of a two-strike “extinction therapy” protocol, in which two treatments are applied sequentially to first contract the population to a vulnerable state and then push it to extinction, and compare it to a combination therapy protocol. We investigate how factors such as the timing of the switch between the two strikes, the rate of emergence of resistant mutations, the dose effects of the applied drugs, the presence of cross-resistance, and whether resistance is a discrete or a quantitative trait affect the outcome. Our results show that the timing of switching to the second strike has a marked effect on the likelihood of driving the cancer to extinction, and that extinction therapy outperforms combination therapy when cross-resistance is present. We conduct an *in silico* trial that reveals when and why a second strike will succeed or fail. Finally, we demonstrate that our conclusions hold whether we model resistance as a discrete trait or as a quantitative, multi-locus trait.

## Author summary

Cancer cells often evolve resistance to treatments during therapy by acquiring specific mutations, leading to eventual relapse and treatment failure. While using multiple treatments in combination can often increase the therapy success rate, carefully timing individual therapies may provide a way to avoid resistance and increase the likelihood of success. One method of understanding the effects of treatment timing on therapy success rate is to model of how cancer cells proliferate, mutate, and respond to therapy. In this study, we use such evolutionary simulations to show that, when applying two treatments in sequence to a population of cancer cells, the timing of switching from the first treatment to the second is an important factor in the ability of the treatments to wipe out the cancer cell population. We also show that this timing approach can lead to better outcomes than applying both drugs at the same time when cancer cells are able to acquire resistance to both treatments in a single mutation. Our work indicates that modeling the evolution of resistance in cancer may enhance the success of existing therapies and reduce the likelihood of relapse through more controlled treatment timing.

## Introduction

Cancer is a heterogeneous collection of diseases with a wide variety of outcomes, natural histories and responses to therapy [1–6]. Cancerous neoplasms are often comprised of a diverse amalgamation of normal and malignant cells. A defining feature of cancerous cells is that they arise, divide, and proliferate uncontrollably as they adapt to their host environment through natural selection [7]. Efforts to characterize the clonal evolutionary dynamics of cancer have been ongoing for decades [8–16]. These efforts include experimental investigation of cancer’s cellular kinetics (growth and death rates) and characterization of genetic changes during initial growth and following treatment. Cancer treatments such as immuno- and chemo-therapeutics pose a strong selective pressure on these populations of cells. Ideally, the effects of these drugs on cancer cell populations are strong enough to drive the cancer cells to extinction, but it is common for these populations to evolve resistance to treatment and then resume uncontrolled growth [17–20]. Resistance to therapy represents a major obstacle to long-term progression free survival of patients. A better understanding of the evolutionary processes of cancer cell populations could lead to therapies that better mitigate or even exploit adaptive responses to treatment [21]. Modeling studies have proved useful for improving our understanding of the dynamics of cancer initiation, growth, and response to therapy [22–28]. Evolutionary models in particular help us understand when and why some treatment strategies will fail, and may inform new strategies [29–33].

The interactions between a population and selective forces or environmental factors can be investigated using evolutionary and ecological models tailored for cancer, including those that describe the evolution of therapeutic resistance (reviewed in [34] and [35]). For example, evolutionary rescue models [36–39] define the patterns and dynamics of a population threatened with extinction due to a change in selection forces and the probability that that population will adapt sufficiently to avoid extinction and then subsequently recover. Evolutionary rescue occurs when a population is being driven towards extinction by unfavorable changes in the selective environment, but then a beneficial allele (or combination thereof) that is sufficient to mitigate or even benefit from this change becomes common in the population soon enough that the population can rebound before extinction. This results in a U-shaped trajectory of population size that is the hallmark of evolutionary rescue: initial rapid decline to a nadir, followed by renewed expansion [39].

Devising cancer treatment strategies with sustained effectiveness is in part tantamount to finding ways to prevent evolutionary rescue. In cancer, the population consists of the tumor cells, the selective force driving the population towards extinction is the therapy, and the beneficial allele is the mutation that confers drug resistance. Whether a tumor cell population in decline due to treatment will experience rescue depends on the chance that a resistant clonal subpopulation is established soon enough to prevent extinction. This in turn depends on the size of the cancer cell population at the onset of treatment, the rate at which the treatment kills cancer cells (which together determine how long it will take the cancer population to go extinct unless rescue occurs), and the mutation rate for resistance alleles. Higher mutation rates increase the probability that rescue will occur at any point in time. The presence of preexisting resistance mutations also increases the probability of evolutionary rescue [37]. Thus, preventing evolutionary rescue demands therapeutic regimes that maximize the selection against non-resistant tumor cells, while minimizing or delaying mutational events that give rise to resistance. For example, evolutionary rescue theory implies that combining multiple therapeutic drugs with independent modes of action can greatly improve patient outcomes because the rate of appearance of cells resistant to each drug in combination would typically be far lower than the mutation rate for resistance to a single drug. This notion is supported by the success of combination therapy in comparison to single therapy protocols [40–45].

An alternative approach is “extinction therapy” [46–49]. Extinction therapy proposes that after a radical change in the environment resulting in a population crash, the population could be driven to extinction by a comparatively minor environmental perturbation that otherwise would not be a major threat. At the onset, extinction therapy resembles standard-of-care treatment: the most effective available therapy is initially used, continuing this treatment as long as it remains effective. Once the cancer cell population size has been dramatically reduced by the first-line treatment, a “second strike” is introduced with a different drug that may be less effective but is sufficient to push the now-threatened cancer cell population to extinction. Evolutionary modeling has shown that this “two-strike” extinction therapy can be effective [47,50], presuming the second strike is not too early (before the population has shrunk enough to be threatened by a less-effective treatment) or too late (once the population has been rescued by resistance to the first treatment and recovered to a large enough size to survive the second treatment).

Evolutionary modeling can answer outstanding fundamental questions about the conditions under which extinction therapy would be a superior approach, and how it should be carried out. Here we ask: how do factors like the mutation rate and the rate at which a therapy kills cancer cells influence the probability that a two-strike treatment will achieve extinction? What is the optimal timing of the second strike? Perhaps most importantly, when will simultaneous combination therapy be more effective than a sequential two-strike therapy, and vice versa?

Using population genetic simulations, we investigate the optimal timing of the second strike in an extinction therapy treatment protocol and compare the probability of extinction achieved by this sequential two-strike therapy with that of combination therapy. Our analysis reveals: 1) interactions between the optimal timing of two-strike sequential therapy, the mutation rate, and the rate at which the drug kills cancer cells; 2) the scenarios in which combination therapy outperforms optimally timed sequential extinction therapy and vice-versa, and 3) the implications of the genetic architecture of resistance (i.e., a discrete trait caused by a single mutation vs. a quantitative trait resulting from the cumulative effect of many mutations) for the optimal timing and effectiveness of a second strike.

## Methods

Several approaches have been used to model the growth, response to therapy, and clonal evolution of cancer. These include mathematical models [51–54], spatial models [55–57], and agent-based models [58–61].

We used the software SLiM 4.0 [62] to simulate a diploid clonal population of cells undergoing expansion, treatment with two drugs, and mutations that confer resistance to these drugs. This software allows for agent-based evolutionary simulations which are widely used in the field of population genetics and provide a great deal of flexibility in modeling the evolution of resistance with minimal mathematical assumptions. Cells in these simulations are diploid and thus can receive resistance mutations on either haploid genome (see “Mutation” below for details on this process). To model clonal evolutionary dynamics, we set the recombination rate to 0. Events in the simulation occur over discrete generations (called cycles in SLiM), with the following events occurring sequentially during each generation (Fig A in S1 Text):

1. Cell replication: Each cell initially undergoes mitotic replication, producing two daughter cells with the exact same genome (i.e., containing the same set of mutations) as the parent cell. Thus, the cancer cell population size doubles during this step.
2. Mutation: After cell division, each daughter cell may acquire new mutations that affect resistance to treatment (see step 3). Mutations randomly occur according to the specified mutation rate, which represents the probability of a resistance mutation appearing anywhere in the genome per cell per cell cycle. The type of a mutation specifies the kind of resistance the mutation confers, with three options: resistance to the first drug only, resistance to the second drug only, and resistance to both drugs (cross-resistance). If a mutation occurs, its type is determined according to a discrete probability distribution of mutation types, referred to as the draw rates. ƒ rate of ${\text{1}\text{0}}^{-\text{5}}$ with draw rates of 0.45, 0.45, and 0.1 for the three mutation types means the first and second type will have a probability of $\text{0}.\text{4}\text{5}\times {\text{1}\text{0}}^{-\text{5}}$, and the third type a probability of $\text{0}.\text{1}\times {\text{1}\text{0}}^{-\text{5}}$ of appearing on each of the two haploid genomes each time a cell undergoes division.
3. Calculation of fitness costs: This step involves tallying the number of mutations in a cell and then calculating its fitness depending on the presence or absence of treatment during the current generation. Treatment reduces fitness, but resistance mutations can mitigate or eliminate this fitness cost. Cells may also die in the absence of treatment (“turnover”).
4. Death and survival: Next, the fitness costs incurred by each cell are used to calculate the probability of survival. At this step, each cell has a probability of survival, $P_{surv}$, while the remaining cells die and thus removed from the simulation. Thus, the population contracts during this step, and cells that survive return to step one (replication) in the next cell cycle.

### First model: two-strike extinction therapy in an evolutionary rescue model

A simulation begins with the expansion phase: a population starting with a single cell grows exponentially as described above until reaching a size of ~${\text{2}}^{\text{2}\text{0}}$. We note that this population size of about one million cells is smaller than the number of cells found in a clinically relevant tumor, but simulating smaller populations is a useful way of modeling the dynamics of larger populations while maintaining computational efficiency (but see [63] for caveats about this type of scaling). Next comes the treatment phase, which consists of the application of the first drug for a specified number of time steps, followed by immediately switching to the second drug. The number of cell cycles during which the first drug is applied is referred to as the “second-strike lag time”.

Throughout the simulation, only mutations that confer resistance to the first drug or the second drug can appear, and no cross-resistance mutations are ever present (although cells can acquire both types of resistance mutations independently). However, these mutations confer no fitness advantage or cost during the expansion and thus evolve under purely random drift throughout this phase. Note that we did not allow back-mutations: once a cell has acquired a given resistance mutation, all its descendants will harbor that same resistance allele.

In this model, resistance is a discrete trait, and resistant cells do not experience any reduction in survival due to treatment nor due to any fitness tradeoff associated with resistance. The magnitude of the dose effect of each drug, quantified as the probability that any given cell will be killed by that drug, is a tunable parameter; we adopt the shorthand “dose” to refer to this parameter in our model. The equation governing survival probability during step 4 of each generation is as follows:


$$\begin{array}{c} P_{surv}=\left(\text{1}-r_{turnover}\right)\times \left(\text{1}-dose_{drug_{\text{1}}}\times \left(\text{1}-{\text{1}}_{m\text{1}}\right)\right)\times \left(\text{1}-dose_{drug_{\text{2}}}\times \left(\text{1}-{\text{1}}_{m\text{2}}\right)\right) \end{array}$$

where:

$r_{turnover}$ is the turnover rate of cells. We set this to 0.20 for all simulations (i.e., 80% of cells survive in the absence of treatment)

$dos{e_{drug}}_{\text{1}}$ is the dose of the first drug,

$dos{e_{drug}}_{\text{2}}$ is the dose of the second drug,

${\text{1}}_{m\text{1}}$ is the indicator function that the cell has a mutation resistant to drug 1,

and ${\text{1}}_{m\text{2}}$ is the indicator function that the cell has a mutation resistant to drug 2. In this discrete-trait model, if a mutation of a given type occurs in a cell that already has a mutation of that type, there is no change to the cell’s resistance phenotype. As a consequence, all resistance mutations are dominant and thus there would be no additional effect of having a second copy of the resistance allele (which can only occur if a site is mutated independently on both haploid genomes since our model does not allow any form of recombination).

During the expansion phase, both drug doses are zero, meaning fitness is only governed by the turnover rate. Application of either drug entails setting its dose to a specified value > 0 while the other drug’s dose remains at zero. The effect of each drug’s dose is to reduce the probability of survival, and the indicator resistance functions govern whether a cell will experience this reduction in survival probability. Note that we use the shorthand “drug” to refer to each treatment, but each of these treatments could themselves be thought of as a combination of multiple drugs or other therapeutic agents that together kills cancer cells according to the specified dose parameter and to which resistance evolves at the specified mutation rate.

We note that this simulation model differs from that of Gatenby et al.’s [47] extinction therapy simulations in several key respects: 1) We model the two strikes in the same manner in that they are continually applied drugs that each reduce the fitness of cancer cells, while Gatenby et al. modeled the second strike as an instantaneous 40% reduction in the cancer cell population size rather than a continually applied treatment; 2) We end treatment with the first drug at the moment the second strike begins, whereas Gatenby et al.’s model continues therapy 1 after the second strike; 3) Gatenby et al. modeled resistance as a quantitative rather than discrete trait (a change we experiment with below); 4) Gatenby et al.’s model includes an Allee effect, while ours does not; and 5) We assume that all birth and death events are synchronous, whereas agent-based models of cancer cell populations (such as Gatenby et al.’s model) often assume asynchronous birth and death events, a difference that may affect the variance in simulation dynamics [64].

For our first set of simulations, we varied the rate of mutation to resistance alleles and the second-strike lag time to test the effect of these parameters on treatment success. We tested per-genome resistance mutation rates between $\text{2}.\text{0}\times {\text{1}\text{0}}^{-\text{6}}$ and $\text{6}.\text{2}\times {\text{1}\text{0}}^{-\text{5}}$ with increments of $\text{4}\times {\text{1}\text{0}}^{-\text{6}}$ and second-strike lags between 1 and 20 cell cycles with increments of 1 cell cycle. Our drug dose effects in these simulations were fixed at 0.75 for both drugs (i.e., 75% of sensitive cells are killed by the drug each generation, on average). We ran 100 replicates of each parameter combination and measured the fraction of replicates in which the population went extinct, which we refer to as the “fraction of extinct replicates.” We also tested the effect of using a lower dose of either drug across the various mutation rates. Furthermore, we fixed the mutation rate and examined the effect of varying both drug doses simultaneously.

### Fixed-trajectory simulations for *in silico* trials

In addition to examining sets of independent simulation replicates, we also conducted an *in silico* trial examining how outcomes of two-strike extinction therapy differed among a set of 50 *in silico* patients, where each patient is defined by a combination of mutation rate and drug dose. Specifically, under a variant of our first model, we examined the dynamics leading to extinction or evolutionary rescue for each patient by fixing the trajectory of the population up to the time of applying the second treatment—this was achieved by using a fixed initial random seed that was unique to that patient. When the second strike happens, a new replicate-specific random seed is chosen. This means that up until the time of the second strike, there is stochastic variation between the patients, but not between replicates within one patient. After the second strike, there is stochastic variation in the population trajectory both between replicates of the same patient and between all patients. These trajectories include all birth and death events and all mutations. We also carried out simulations where a second strike never occurs. In these simulations, the population is allowed to rescue without ever switching treatments. Since no second strike occurs, and since stochasticity between replicates only occurs after the second strike, these simulations have a completely fixed trajectory for each patient. We use these simulations to observe the complete trajectory of population size and the frequency of resistance mutations and examine how these dynamics affect the probability of extinction in those simulation replicates where a second strike does occur.

### Second model: The effect of combination therapy

We compared a combination therapy consisting of the simultaneous application of both drugs to sequential two-strike extinction therapy, again considering a range of second-strike lag times for the latter. In these simulations, combination therapy is simulated as both drugs having constant doses higher than zero throughout the treatment phase. The dose effects of both drugs in these simulations are always equal, and the lowest dose used for each drug in the combination was 0.4. We note that when both drug doses are 0.4, the survival probability of cells is still below 0.5 (see survival probability formula in Methods), meaning that a net decrease in population size is expected in the absence of resistance. For the two-strike simulations included in this comparison, we fix the dose effect of both drugs to 0.9, while varying this dose effect during combination therapy. We ran 100 replicates of each parameter combination.

### Third model: Cross-resistance

We modified the second model to include a third rare mutation type which confers resistance to both drugs. The draw rates for m1, m2, and m3 mutations under this third model are 45%, 45%, and 10% respectively. The survival probability under this model is:


$$\begin{array}{cc} P_{surv}=\left(\text{1}-r_{turnover}\right)\times \\ & \text{\textasciitilde{}}\left(\text{1}-\left(dose_{drug_{\text{1}}}\times \left(\text{1}-{\text{1}}_{m\text{1}}\right)\times \left(\text{1}-{\text{1}}_{m\text{3}}\times strength_{cross_{resistance}}\right)\right)\right)\\ & \times \left(\text{1}-\left(dose_{drug_{\text{2}}}\times \left(\text{1}-{\text{1}}_{m\text{2}}\right)\times \left(\text{1}-{\text{1}}_{m\text{3}}\times strength_{cross_{resistance}}\right)\right)\right) \end{array}$$

This equation shows that the presence of cross-resistance mutations (i.e., ${\text{1}}_{m\text{3}}=\text{1})$ dampens the effect of both drugs on fitness in proportion to the strength of the cross-resistance. Here, a cell that lacks a mutation of type m1 or m2 can still be resistant to both drugs if it has a mutation of type m3, but rather than full resistance, the degree of resistance imparted by this mutation is determined by $strength_{cross\_resistance}$; if $strength_{cross\_resistance}=\text{0}$ then m3 provides no resistance, but if $strength_{cross\_resistance}=\text{1}$ then m3 confers full resistance to both drugs. We ran these simulations across a range of values of $strength_{cross\_resistance}$, comparing the outcomes of combination therapy to sequential therapy with various lag times.

### Fourth model: Varying drug doses and the mutation rate ratio in combination therapy

To investigate whether the optimal combination of doses during combination therapy depends on the relative rates of mutation to resistance alleles for the two drugs, we carried out simulations with varying ratios of the rates of m1 and m2 mutations (achieved by varying the rate of m1 mutations while fixing the rate of m2 mutations). Specifically, we let the mutation rate for m1 mutations range from ${\text{1}\times \text{1}\text{0}}^{-\text{5}}$ to $\text{1}.\text{0}\text{1}\times {\text{1}\text{0}}^{-\text{3}}$ while the rate for m2 mutations was fixed to $\text{1}.\text{5}{\times \text{1}\text{0}}^{-\text{5}}$. We also varied the ratio of the doses of the two drugs, under the constraint that $\left(\text{1}-dose_{drug_{\text{1}}}\right)\times \left(\text{1}-dose_{drug_{\text{2}}}\right)$ is fixed at 0.25. This was done by setting $dose_{drug_{\text{1}}}$ to a value between 0.30 and 0.70 and then setting $dose_{drug\text{2}}$ as follows:


$$\begin{array}{c} dose_{drug_{\text{2}}}=\text{1}-\text{0}.\text{2}\text{5}/(\text{1}-dose_{drug_{\text{1}}}) \end{array}$$

This ensures that the probability of survival for non-resistant cells is the same across all parameter combinations for this model. Any differences in extinction probability across parameterizations are therefore due to the interplay between drug mutation rates and drug doses.

### Simulations modeling resistance as a quantitative trait

We also experimented with a model where drug resistance is a quantitative trait [36]. Cells can accumulate mutations conferring resistance to either drug. Each mutation contributes additively to resistance against the corresponding drug and its contribution is drawn from a lognormal distribution. The parameters of this distribution were chosen so that the average resistance effect of a new mutation was 0.1, meaning that, on average, 10 accumulated mutations of a specific type should confer full resistance to the corresponding drug. This yielded a mean of -2.42759 and a standard deviation of 0.5. The survival equation then becomes:


$$\begin{array}{c} P_{surv}=\left(\text{1}-r_{turnover}\right)\times \left(\text{1}-dose_{drug_{\text{1}}}\times \left(\text{1}-\sum_{i}W_{m\text{1},i}\right)\right)\times \left(\text{1}-dose_{drug_{\text{2}}}\times \left(\text{1}-\sum_{i}W_{m\text{2},i}\right)\right) \end{array}$$

where:

$r_{turnover}$, $dos{e_{drug}}_{\text{1}}$, and$dos{e_{drug}}_{\text{2}}$ are as defined above,

$W_{m\text{1},i}$ is the fitness effect of the i^th^ m1 mutation (zero if no such mutation present), and $W_{m\text{2},i}$ is the fitness effect of the i^th^ m2 mutation, if present. Thus, $\sum_{i}W_{m\text{1},i}$ and $\sum_{i}W_{m\text{2},i}$ represent the cell’s total amount of resistance to drugs 1 and 2, respectively

Note that under this model mutations can only increase resistance, and the combined effect of all resistance mutations in a genome was capped at 1. In addition, we emphasize that the effects of all resistance mutations are fully additive, and this extends to those rare cases where a mutation occurs at the same site on both haploid genomes—in such cases the two mutations are treated as independent (i.e., their effects are drawn independently from the lognormal distribution described above, and then summed together along with those of any other resistance mutations present in the cell). Thus, the only consequence of having diploid rather than haploid cells in both our discrete and quantitative trait models is that the total mutation rate is effectively doubled.

### Use of Artificial Intelligence

Anthropic’s Claude Code utilizing Sonnet 4.6 was used to refactor code for this study during the revision process. The refactored portions of the code were responsible for aggregating data from simulations and producing figures. All generated code was thoroughly reviewed, and its output was compared against the output of the original code for validation.

## Results

### Our model: Progression after treatment as a case of evolutionary rescue

Typical cancer treatment involves applying the maximum tolerated dose (MTD) of a first-line therapy until either the cancer is eradicated or until progression is observed [65]. Progression often occurs because of the emergence and proliferation of treatment-resistant cells within the cancer cell population. We model this as a form of evolutionary rescue (Methods). A typical trajectory of a population undergoing evolutionary rescue in our model is shown in Fig 1. In this example, the population size is initially growing exponentially, but then rapidly declines following the application of treatment. Eventually, the population size decreases to such a low level that it may be undetectable. However, when the population is approaching its nadir, a subpopulation of resistant cells emerges and begins to become more prevalent, ensuring that the treatment will not succeed in eradicating the cancer.

**Fig 1:**
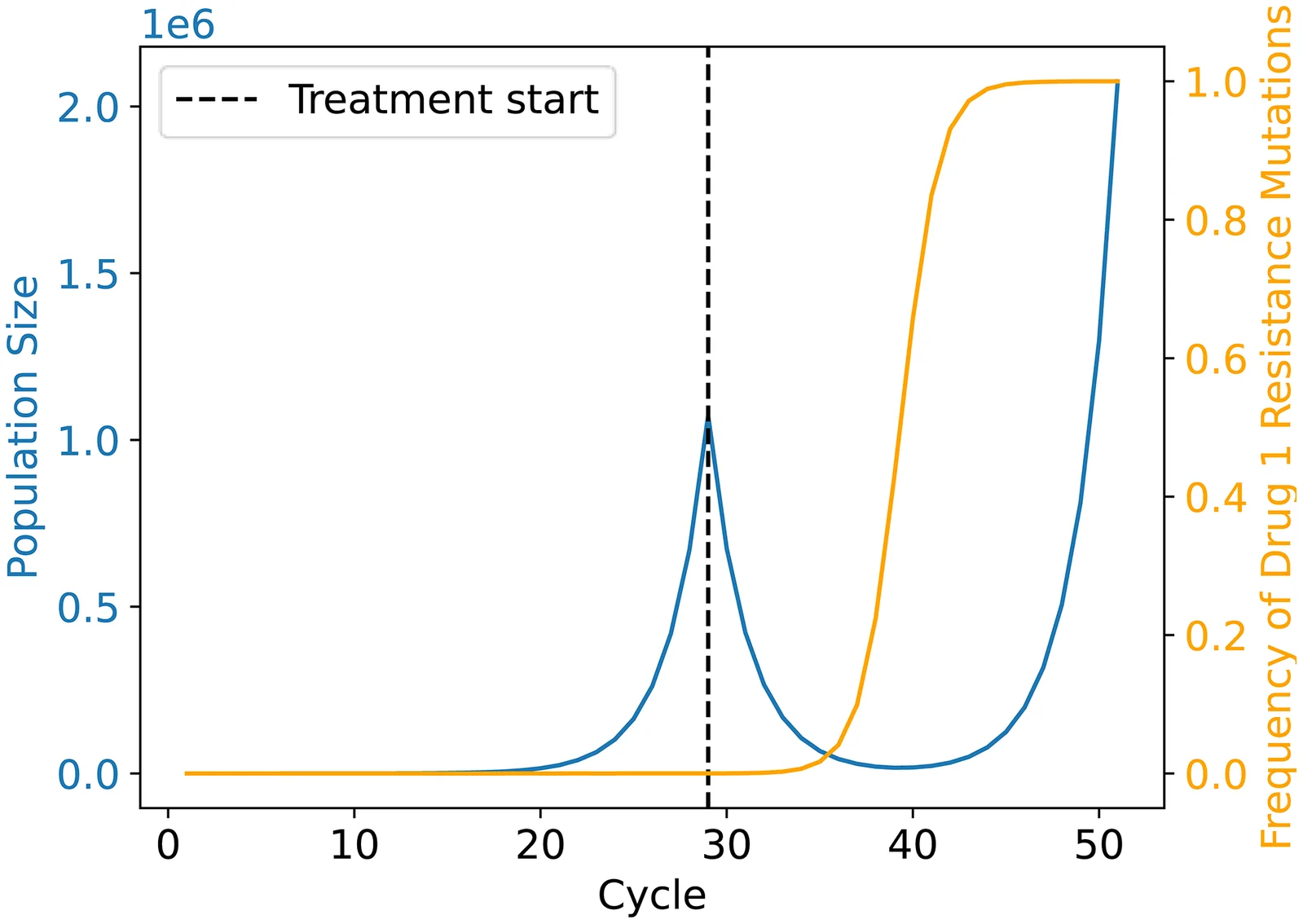
Trajectory of a cancer cell population during evolutionary rescue following treatment. The population expands exponentially (blue line) until the first strike therapy is applied (dashed black line), after which the population declines rapidly. However, as the population is declining, a resistance mutation has emerged and begun to increase in frequency in the population. As the treatment continues, the frequency of the resistance mutation continues to increase (orange line), the population eventually becomes fully resistant, resumes its exponential expansion, and ultimately recovers and progresses beyond its initial size pre-treatment. This is the classic U-shaped curve of evolutionary rescue. This example trajectory is taken from a single simulation replicate of our first model (Methods), but without the application of a second strike. For this replicate the mutation rate was set to $\text{1}.\text{5}{\times \text{1}\text{0}}^{-\text{5}}$ and the drug dose to ~0.61 (indicating that the fitness of treatment-sensitive cells is multiplied by ~0.39 during treatment; see Methods).

Differences in treatment regime can alter the trajectory of evolutionary rescue. Below we assess the efficacy of alternative treatment strategies that are designed to reduce the probability of evolutionary rescue of the cancer cell population.

### Investigating the dynamics of evolutionary rescue in extinction therapy via in silico trials

#### Extinction therapy succeeds when the first strike removes variants that confer resistance to the second strike.

Our simulation approach allows us to ask, for a given simulated patient’s disease course, what would have been the optimal treatment strategy? Such information would not only improve our understanding of the dynamics impacting the probability of extinction but also help us assess the potential of treatment strategies tailored to individual patients. To this end, we conducted an *in silico* trail with a set of patients receiving two-strike extinction therapy, and determined, for each specific patient, the optimal timing of the switch to the second strike (the “second-strike lag time”). This may differ from patient to patient both because parameter values such as the mutation rate or drug dose effect (“dose” for short) differ across patients, and because of stochasticity in the timing of the emergence of resistance and other random events occurring both before and during the treatment course.

Our trial consisted of a set of 50 *in silico* patients, each of whom experienced a deterministic disease trajectory up until the start of the second strike, at which point we performed a set of 100 stochastic simulations to observe that patient’s distribution of outcomes for a given lag time (Methods). Illustrative example patients are shown in Fig 2; the remainder are available on our project’s GitHub repository. An examination of these results reveals that two different, but related factors govern the probability of treatment success (i.e., the fraction of extinct replicates) for a given lag time. The first is the size of the population of cancer cells at the time of the second strike, and second is the frequency of the mutations at that time—results that are consistent with analytical results [37]. We might expect the optimal lag time to be the moment the population size is at its minimum (*t*_*min*_), as a relatively small number of additional cell deaths are needed to result in extinction, and indeed in many cases we see that the extinction probability peaks when the second strike begins at *t*_*min*_. Two such examples are shown in Fig 2C and 2D; for these two synthetic patients a plateau of extinction probability begins (green curves) when the second strike starts at *t*_*min*_.

**Fig 2:**
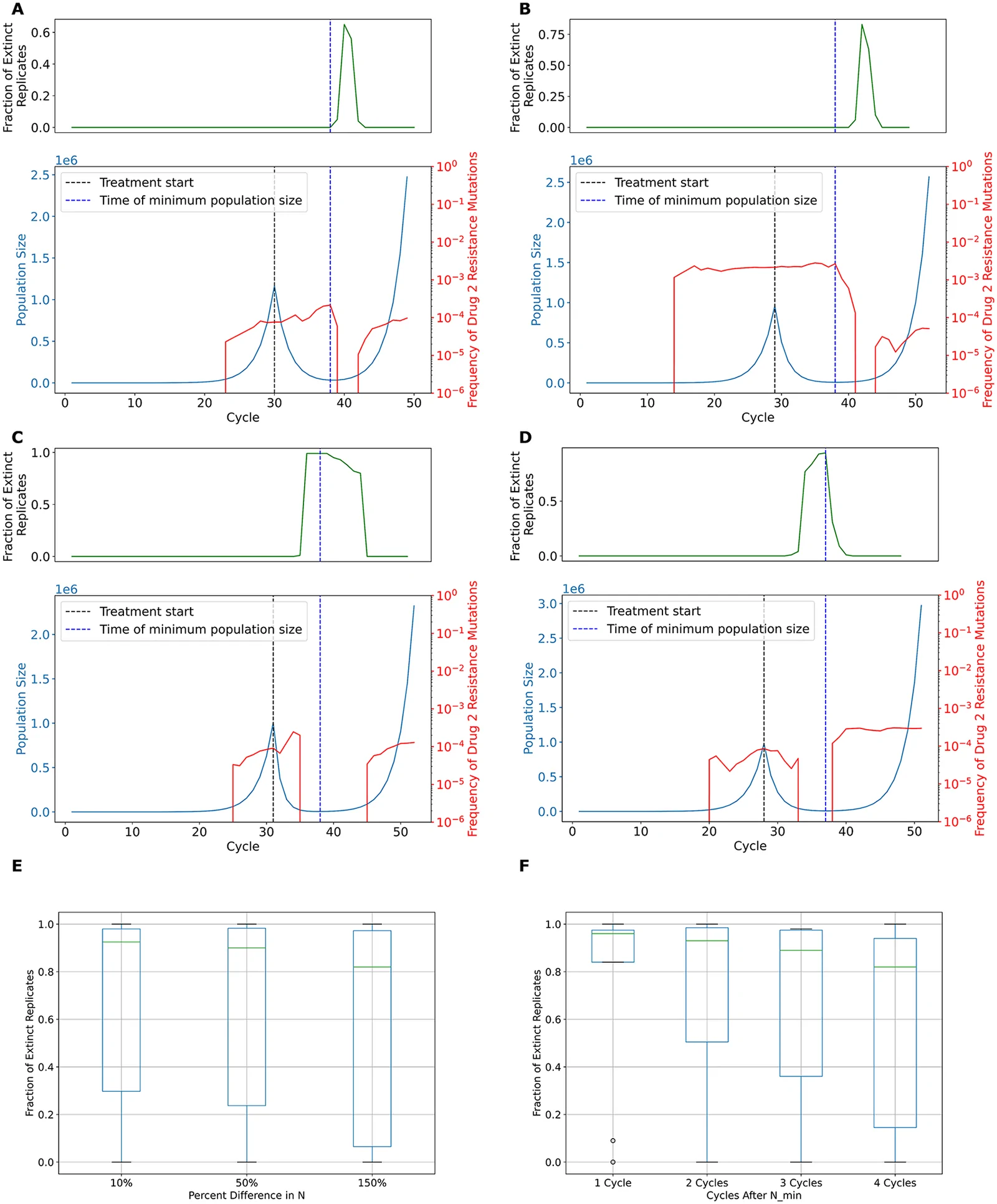
Synthetic patient trajectories show the success of strategies that involve switching treatments near the population nadir and the importance of eliminating standing variation. Panels (A-D) show, for four in silico patients, the extinction probability when the second-strike occurs at each cycle of the simulation following the first strike (dashed black line), and how this depends on the population size and the presence of standing variation conferring resistance to the second strike. In these panels, the population size (blue line) and the frequency of mutants resistant to the second strike (red line) are from simulations which experience no second strike. These lines therefore represent the deterministic trajectory of that patient in the absence of a second strike. Green lines in the upper sub-panels represent the fraction of replicates in which the cancer cell population experienced extinction when the second strike began at that cycle; recall that stochasticity between replicates only occurs after the second strike starts (Methods). A-B) Trajectories of synthetic patients where the optimal lag time occurs after the time of minimum population size (dashed blue vertical line) due to remaining standing variation that confers resistance to the second treatment. C-D) Trajectories of synthetic patients where the optimal lag time is at the point where the population reaches its minimum size because standing variation that confers resistance to the second treatment has been eliminated prior to the population reaching its minimum. Results for the remaining 46 in silico patients are available on our GitHub repository. E) The distribution of extinction probabilities for a separate set of validation patients across three different timing strategies for the second strike. Each value on the *x*-axis shows the percent growth in *N* (population size) relative to *N*_*min*_ (the size of the minimum population at the time of switching from the first strike to the second strike). F) The distribution of extinction probabilities for the set of validation patients across three more timing strategies for the second strike; each value on the *x*-axis shows the number of cycles allowed to occur after reaching *N*_*min*_.

However, beginning the second strike at *t*_*min*_ is not the optimal strategy for all synthetic patients. This is because in some cases there is standing variation for resistance to the second treatment present at *t*_*min*_. Although they confer no fitness benefit prior to the second strike, resistance mutations for the second drug typically emerge during the expansion phase and persist at low frequency; sometimes they remain present even as the population declines to its minimum size (see Fig 2A and 2B for two such examples). In such cases, beginning the second strike at *t*_*min*_ will almost always fail to achieve extinction. Instead, continuing the first treatment for one or more cycles beyond *t*_*min*_ may reduce the frequency of these resistance mutations or even remove them from the population, resulting in a much higher extinction probability (i.e., the extinction probability in Fig 2A, 2B peaks only after the fraction of cells resistant to drug 2 (referred to as f below), shown via red curves, has plummeted). This makes sense because, during the first strike, cells that are resistant to the first treatment are selected for at the expense of others, including those that have preexisting resistance to the second treatment, thereby reducing the chance of evolutionary rescue following the second strike (e.g., Fig 2A and 2B).

#### *In silico* trials inform strategies for timing the second strike.

Our synthetic trials also identify two-strike treatment strategies that are more likely to prevent evolutionary rescue of the cancer cell population. For example, when examining the results for all 50 synthetic patients, we find that switching to the second treatment at the time where the population is at or near its minimum after the first strike is generally a successful strategy (e.g., when the cancer cells’ mutation rate is set to $\text{2}.\text{5}\times {\text{1}\text{0}}^{-\text{5}}$, the strategy of beginning the second strike at *t*_*min*_ results in an extinction probabili*t*y that is within 3% of its maximum for ~70% of synthetic patients).

In practice, however, it may not be possible to known *t*_*min*_, until after the population has begun to rebound. We therefore assessed the effectiveness of beginning the second strike after some amount of time/growth after *t*_*min*_ is observed. First, we assessed the effectiveness of beginning the second strike when the cell population has grown either 10%, 50%, or 150% larger than its minimum size following application of the first strike. This generally results in a high extinction probability (Fig 4E), but this probability decreases from >90% to ~80% as we increase the amount of growth that we allow to occur between *t*_*min*_ and *t*he beginning of the second strike from 10% to 150%); the variance in outcomes also increases with the amount of lag time post-*t*_*min*_. Similarly, applying *t*he second strike after a specific number of cycles (i.e., 2, 3, or 4 cycles) after *t*_*min*_ again yields generally good resul*t*s (Fig 2F), although again efficacy decreases and outcome variability increases as the amount of post-*t*_*min*_ lag *t*ime before the second strike increases. In summary, our results suggest that while there may be variation in outcomes from patient to patient, a second strike is most likely to be successful if it can be initiated at or shortly after *t*_*min*_.

### The optimal second-strike lag is weakly correlated with the mutation rate

The results from our *in silico* trials demonstrate that the timing of the change from the first-line to the second-line therapy may profoundly affect treatment success [47]. We therefore set out to more closely examine the relationship between second-strike lag time and the probability that the cancer cell population will be driven to extinction. We began by using simulations to estimate the probability of extinction across different second-strike lag times and different rates of mutation to treatment-resistance alleles. Our results (Fig 3A) confirm that there exists an optimal lag time between the onset of the first and second strikes for maximizing the extinction probability of the cell population. In general, the probability of extinction is low when the second drug is applied immediately following the application of the first (a lag time of only 1 cell cycle), then increases with greater lag times until an optimum is reached and then decreases with increasing lag time. While the mutation rate has a weak effect on the optimal lag time, which remains generally between 7 and 9 cell cycles in our model but decreases slightly as the mutation rate increases, it exerts a stronger effect on the dynamics of population extinction probabilities across lag times. For instance, at lower mutation rates the extinction probability exhibits a broad plateau around the optimal lag time, implying that missing the optimal lag time is somewhat forgiving when resistance alleles do not frequently arise. As the mutation rate increases, however, the difference in extinction probability between the optimal lag time and suboptimal becomes starker, with a sharp peak around the optimum, and the extinction probability is also lower in general for all lag times. Another strong effect of the mutation rate is decreasing the extinction probability at the optimal lag time (Fig 3B).

**Fig 3:**
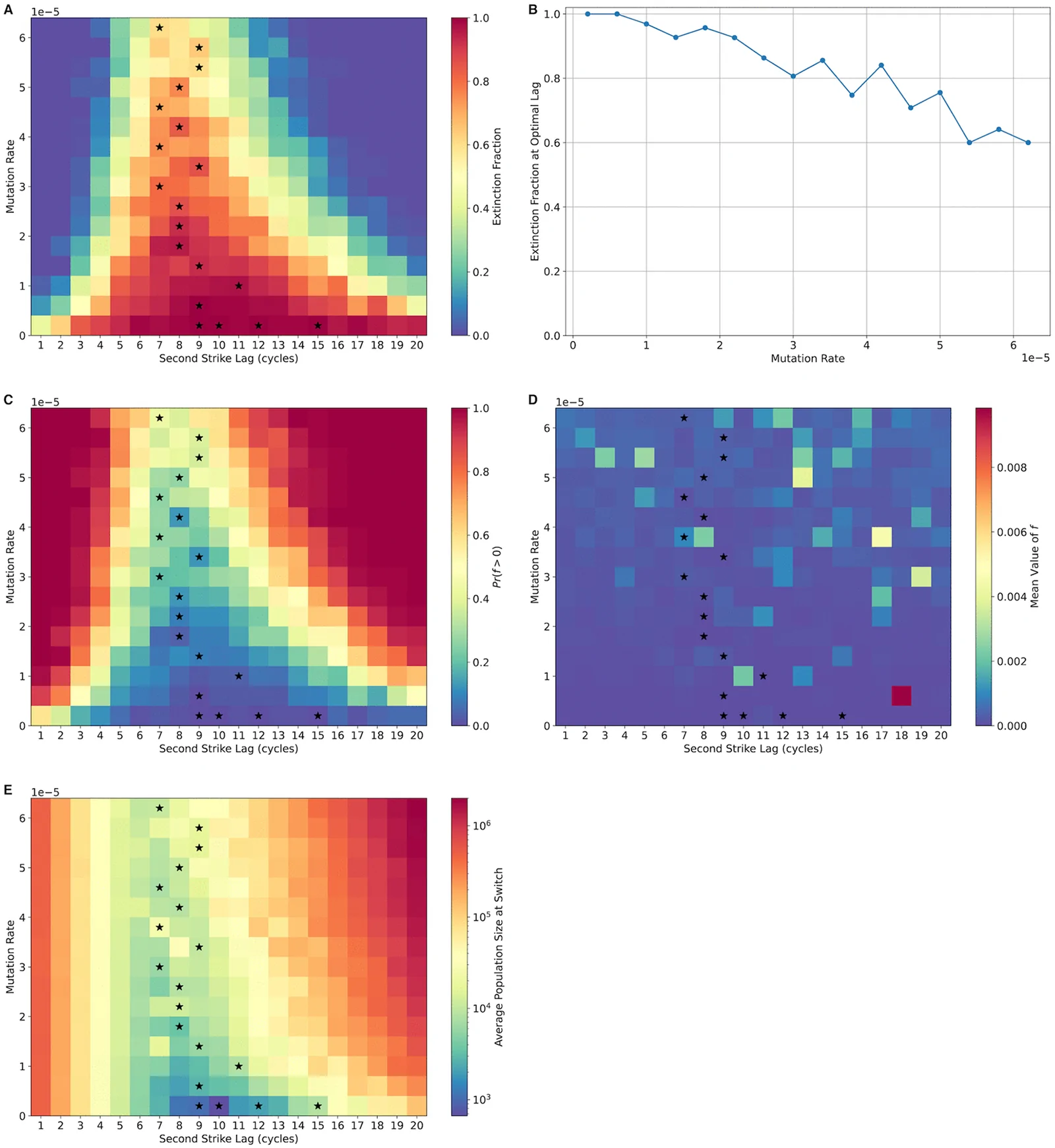
Extinction probability and optimal lag time are largely governed by the probability that mutations conferring resistance to the second drug are already present at the time of the second strike. A) Extinction rate vs mutation rate and lag time. The *x*-axis (Second Strike Lag) represents the time spent under the first treatment before switching to the second treatment. The *y*-axis shows the per-genome mutation rate of resistant mutations. Color indicates the extinction probability estimate and black stars indicate the optimal lag time(s) for each mutation rate—multiple stars are drawn in the case of a tie. B) The mutation rate vs the extinction probability at the optimal lag time for that mutation rate. As the resistance mutation rate increases, the extinction probability decreases. C) The fraction of replicates harboring clones resistant to the second strike immediately before switching treatments (Pr(f>0)) across second-strike lags and mutation rates. Black stars indicate optimal lag time(s). Sharp valleys appear clearly, especially at higher mutation rates, and correspond to the optimal lag time for each mutation rate, suggesting that a low probability that the cancer cell population already contains *any* resistance to the second strike right before switching is what produces the peaks in extinction probability around the optimal lag time. D) The average value of fright before switching treatments for each second-strike lag and mutation rate. Black stars are overlayed from panel A to indicate optimal lag time(s). In general, fseems largely unaffected by the lag time or mutation rate and does not appear to be predictive of the optimal lag time. E) The size of the cancer cell population immediately before switching treatments across second-strike lags and mutation rates. The correspondence between population size and the peak in extinction probability is stronger than that for f (panel D), but not as strong as for that between the extinction probability and the Pr(f>0) (panel C). For all simulations shown in this figure the dose parameter was set to 0.75 for both drugs.

### Treatment success is largely determined by the presence of pre-existing resistance to the second strike

Our *in silico* experiments above suggest that the probability of extinction may depend on whether there is standing variation conferring resistance to the second strike by the time the treatment switch happens. If this were indeed the case, we would expect that the peak in extinction probability observed around the optimal lag time to correspond with a sharp valley of second-strike resistance standing variation. Our results confirm this intuition: while the average frequency of mutants resistant to the second strike (f) measured just before switching to the second strike is not especially predictive of extinction probability or the optimal lag time (Fig 3D), we do observe a sharp valley in the fraction of replicates that have *any* amount of standing resistance variation around the optimal lag time (Fig 3C); we refer to this quantity as our estimate of Pr(f>0). In other words, even though fdoes not markedly change with mutation rate or second-strike lag, Pr(f>0) does, and this appears to be the cause of the sharp peaks in extinction probability seen around the optimal lag times. The population size at the time of the second strike also shows some correspondence with the optimal lag time (Fig 3E), but the relationship is not as strong as for the presence of any pre-existing resistance to the second treatment. The importance of Pr(f>0) for treatment outcomes is underscored by the fact that the Spearman correlation between the probability of extinction and our estimate of Pr(f>0) is -0.996. By contrast, the correlation between extinction probability and the mean value of fis only -0.537, and the correlation between extinction probability and population size is -0.852. In total, for >97% of the simulation replicates shown in Fig 3 in which evolutionary rescue occurred, standing resistance variation was present at the time we switched to the second drug, implying that extinction is unlikely unless this resistance variation is eliminated prior to the second strike.

At first glance it might seem somewhat unexpected that the probability of extinction can be predicted with much greater power by Pr(f>0) than by the average value of f. However, an examination of the data in Table A in S1 Text, where we bin simulations replicates and their outcomes by their value of fat the optimal timing of the second strike ($f_{to}$), clarifies that there are two explanations for this result. First, although the exact value of fdoes affect the probability of extinction, in the vast majority of replicates where we observe pre-existing resistance to the second drug, it is at high enough frequency to essentially guarantee that rescue will occur. Second, even in the minority of cases where the frequency of resistance is lower and evolutionary rescue is not guaranteed, rescue is still by far the most likely outcome. For example, when the second strike is performed at the optimal time, and $f_{to}$ > 0.001, the extinction probability is 0. When 0 <$f_{to}$ <= 0.001, the extinction probability only increases to 0.06. On the other hand, when $f_{to}$ = 0, the extinction probability is 99%. Thus, in our simulations, if f>0, extinction is very likely, and whenever f=0, extinction is very unlikely regardless of the precise value of f. However, this finding may be in part due to the population size rescaling that we performed in our simulations (see Methods), which prevents us from observing extremely low allele frequencies, especially when the population is near its nadir. Thus, real tumors may often have lower values of fthan those examined here, and thus it is possible that the precise value of ffound in a tumor could indeed have a sizable impact on its extinction probability. However, we can still conclude from our simulations that the presence of resistance mutations whose frequency is not extremely low implies a very high probability of treatment failure.

### Extinction is more likely when the more effective drug is used first

The rate at which a population is being driven to extinction is a key determinant of the probability and timing of evolutionary rescue [37,39]. We examined the effect of our drug dose parameters on extinction probabilities and optimal lag times by testing a scenario with a lower dose of drug 1 than drug 2, and one with a lower dose of drug 2 than drug 1. In this context, drug doses control the reduction in fitness for non-resistant cells during the application of the drug (see Methods for fitness equation). We note that the results shared in this section may be altered when there is a fitness cost of drug resistance, something we have not included in our model [50].

When the first drug has a dose of 0.65 and the second has a dose of 0.75, the optimal lag time shifts to around 10 generations (Fig 4A), versus 7–9 generations in the equal-dose effect scenario shown in Fig 3. Again, the mutation rate appears to have a small effect on this optimal lag time, and extinction probabilities are lower for all mutation rates compared to the case where both drugs have a dose of 0.75.

**Fig 4:**
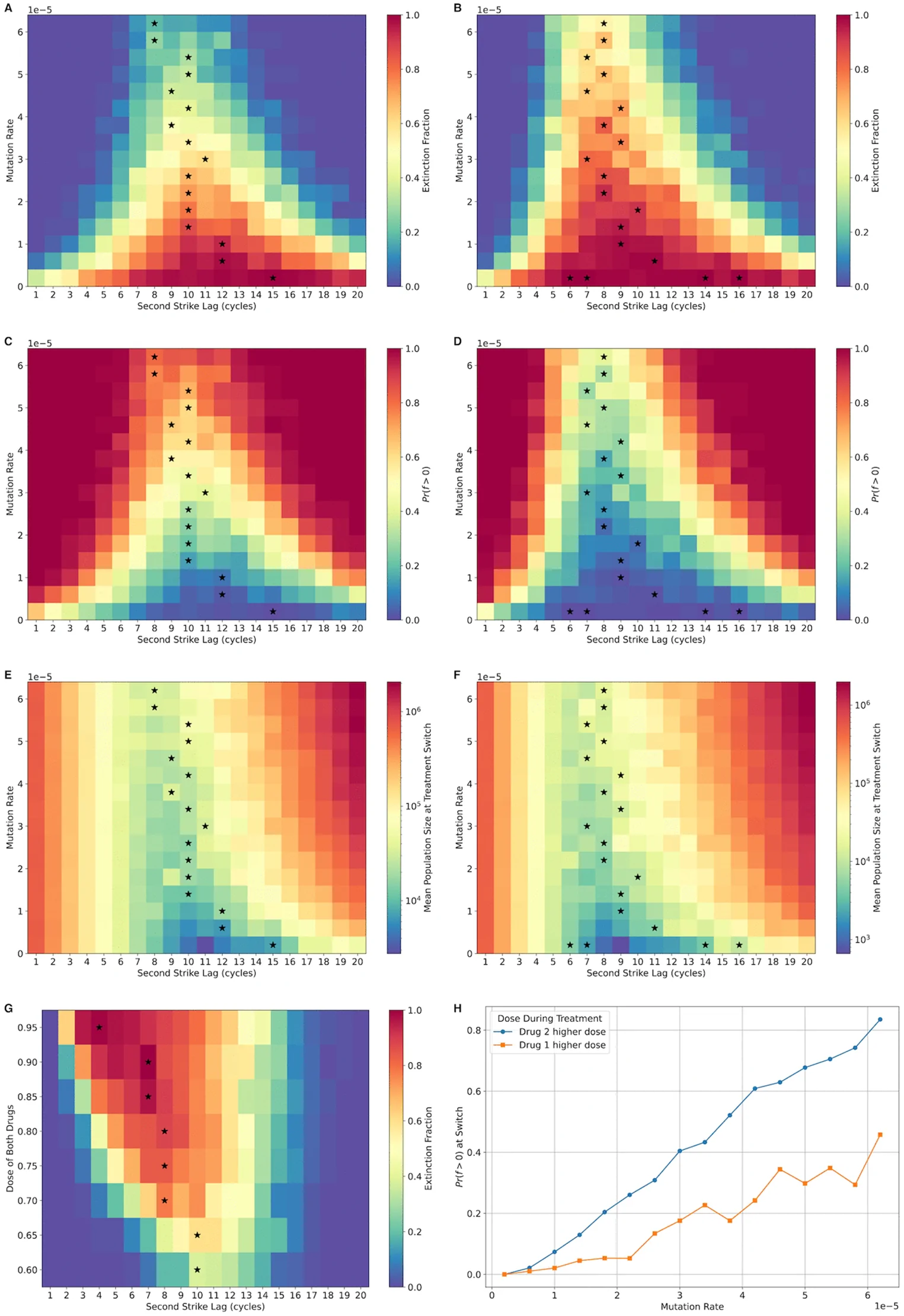
Using a lower-dose drug first reduces overall treatment success and shifts the optimal lag time higher. A) Extinction probability vs mutation rate and lag time when the first drug has a lower dose (0.65) before switching to a second drug with a higher dose (0.75). B) Extinction probability vs mutation rate and lag time when the first drug has a higher dose (0.75) followed by treatment with a second drug with a lower dose (0.65). C) Pr(f>0)immediately before switching treatment for various mutation rates and lag times when the first drug has a lower dose. This fraction is highly predictive of extinction probability and *t*_*min*_ and generally increases with the muta*t*ion rate. D) Pr(f>0) immediately before switching treatment for various mutation rates and lag times when the first drug has a higher dose. Again, Pr(f>0) is highly predictive of extinction probability. E) Average population size right before the second strike when the first drug has a lower dose. This fraction is not as predictive of extinction as is the probability that resistance variation is already present just before the second strike. F) Average population size right before the second strike when the second drug has a lower dose. G) Extinction probability vs lag time and the dose of both drugs when the mutation rate is fixed at $\text{1}.\text{5}{\times \text{1}\text{0}}^{-\text{5}}$, with the dose always being equal for both drugs. Black stars represent the optimal lag time(s) at each mutation rate. H) Pr(f>0), estimated right before the optimal lag for each mutation rate. Blue lines indicate simulations where the second drug has a higher dose and orange lines indicate simulations where the first drug has a higher dose. Unlike the average frequency of these mutants, the fraction of replicates with at least one resistant clone regardless of frequency is consistently higher for simulations where the second drug dose is higher.

Next, we examined the case where the first drug has a higher dose (0.75) than the second (0.65)—a scenario that is more relevant to standard-of-care and to current thinking about extinction therapy [46], which advocates for using the drug with the highest kill rate as the first-line treatment. We found that optimal lag times in this scenario resemble those for the case in which both drugs had an equal dose of 0.75 and are also not dependent on mutation rate. However, the extinction probabilities across all mutation rates are higher compared to the case of the first drug having the lower dose (Fig 4B vs 4A). For instance, at a mutation rate of $\text{3}\times {\text{1}\text{0}}^{-\text{5}}$, simulations where both drugs have a dose of 0.75 result in an extinction probability of ~81% at an optimal lag time of 7, but this decreases to ~61% when the first drug has a lower dose effect (in this case, at an optimal lag time of 11). On the other hand, reducing the dose effect of the second drug but not the first has hardly any effect on the outcome (extinction probability of ~81%, again with an optimal lag of 7). Thus, our results suggest using the stronger drug as the first strike during extinction therapy provides the best chance of eradicating the cancer cell population under our model.

Finally, we set both drugs to an equal dose and examined extinction dynamics across various dose values (with the mutation rate set to $\text{3}{\times \text{1}\text{0}}^{-\text{5}}$ in each instance). When the dose of both drugs is equal, the optimal time increases as the drug dose decreases, and the extinction probability decreases from ~100% at a drug dose of 0.95 to ~37% at a drug dose of 0.60 (Fig 4G).

Consistent with our previous results, the difference in extinction dynamics between the different strategies examined in this section results from differences in the probability that resistance mutations are present right before switching treatments, but not the frequency of these mutations or the population size. For instance, the average frequency of these resistance mutations (f) in the population again shows little correspondence with the probability of extinction or the optimal lag time in our simulations (Panels A and B in Fig B in S1 Text). Meanwhile, the average population size right before the second strike again shows slightly higher correlation with extinction (Fig 4E and 4F), although the observed valleys around the extinction time remain wide. On the other hand, our estimate of Pr(f>0) at the time of the second strike is highly predictive of both extinction probability and optimal lag time, with sharp valleys around the optimal lag times and a general decrease with increasing mutation rate (Fig 4C and 4D). Consistent with the notion that the probability of any standing resistance variation is more important than the frequency of these variants in the population in driving the difference between the best-drug-first and best-drug-second strategies, we note that as the mutation rate increases the differences in extinction probability between these two strategies increases as well (Fig 4A and 4B). Concomitantly, our estimate of Pr(f>0)at the the optimal time of the second strike) is always higher when using in the best-drug-second strategy than when using the best drug first, and the difference between the two increases with mutation rate (Fig 4H). In contrast, the ratio of the mean value of $f_{to}$ under the best-drug-second strategy to that under the best-drug-first strategy does not consistently increase with mutation rate (Panel S in Fig B in S1 Text). We also note that the time it takes the population to reach its minimum (*t*_*min*_) is higher when the best drug is used second (Panel D in Fig B in S1 Text), and cumulative reduction in population size is less severe (Panel E in Fig B in S1 Text) than when the best drug is used first. In essence, when the dose of the first drug is higher, the popula*t*ion experiences a more rapid and intense population bottleneck than when the dose the second drug is higher, thereby increasing drift and therefore likelihood of having replicates with no standing variation that is able to rescue the population after switching the second drug.

### Combination therapy outperforms sequential therapy, except when cross-resistance is strong

To compare the efficacy of combination therapy to optimally timed sequential therapy, we conducted simulations based on the sequential therapy scenario above but included the possibility of combination therapy. In the latter case, both drugs were applied simultaneously and at an equal dose until population rescue or extinction. This model assumes that such a combination is tolerated and that the drugs do not interfere with each other’s efficacy. We compared these simulations in the presence and absence of the possibility of mutations conferring cross-resistance to both drugs. Our results (Figs 5A and Fig C in S1 Text) show that, in the *absence* of cross-resistance, combination therapy outperforms optimally timed sequential therapy for all combination drug doses of 0.45 and above. As expected, the extinction probability increases with increase in the dose of the combination drugs.

**Fig 5:**
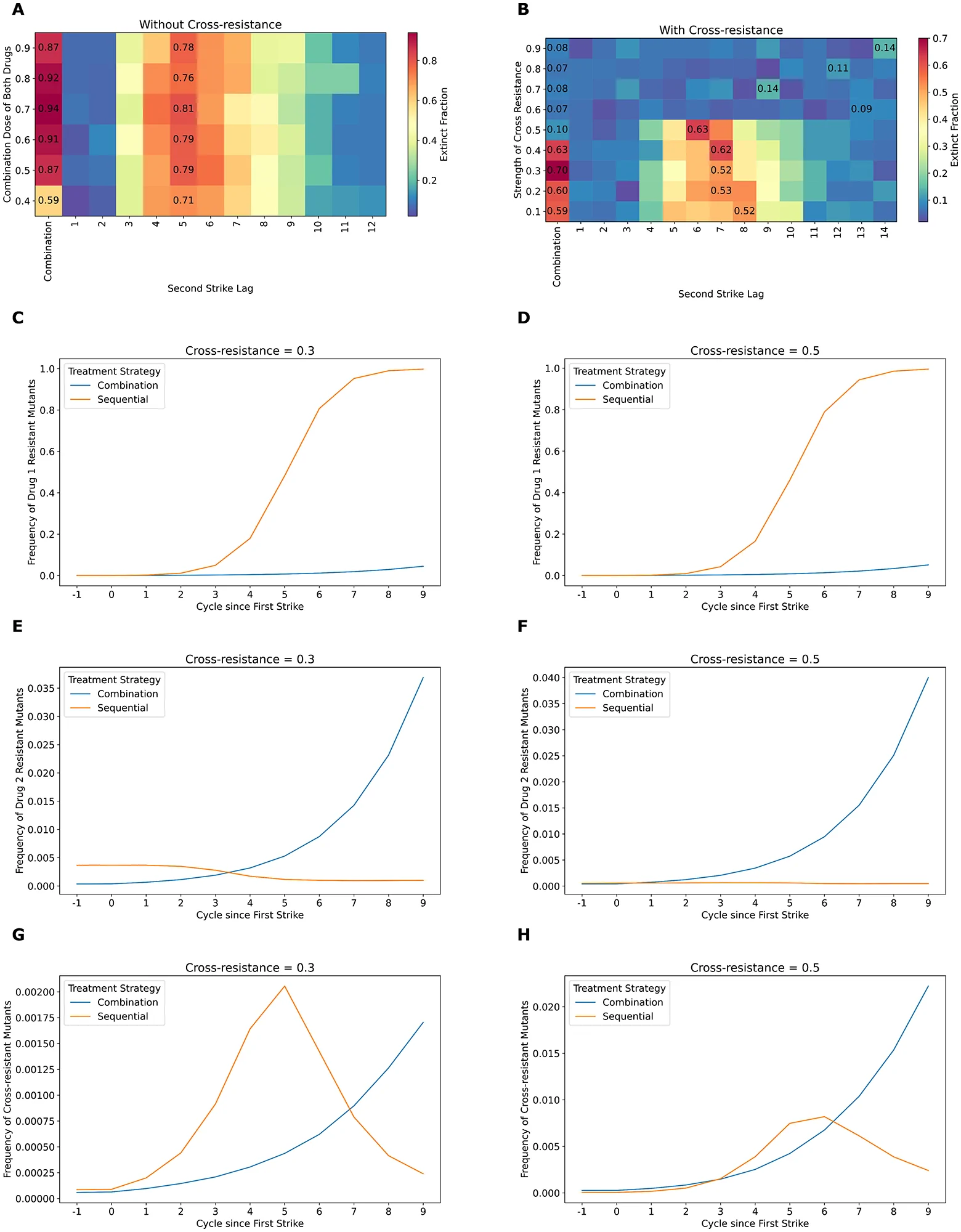
Comparison of efficacy between combination therapy and sequential therapy in the presence and absence of cross-resistance mutations shows that combination therapy is usually more effective except in cases of high cross-resistance. A) Combination therapy and sequential therapy at a mutation rate of ${\text{1}\text{0}}^{-\text{6}}$ in the absence of cross-resistance at various doses of combination drug. The sequential drug doses are fixed at 0.90 for both drugs. Results for additional mutation rates are shown in Panel B in Fig C in S1 Text. B) Combination therapy and sequential therapy at a mutation rate of ${\text{1}\text{0}}^{-\text{6}}$ at various strengths of cross-resistance; the combination drug doses are fixed at 0.4 per drug and the sequential drug doses are fixed at 0.8 for both drugs. The fraction of extinct replicates for the sequential protocol at the optimal time and for the combination protocol are indicated in black text. Results for additional mutation rates are shown in Panels C – H in Fig D in S1 Text. C-H) The average frequency of the three types of resistant mutants across all replicates a few cycles after the beginning of treatment during both sequential (orange lines) and combination (blue lines) treatment with a mutation rate of ${\text{1}\text{0}}^{-\text{6}}$. Each pair of panels depicts mutant frequency trajectories for a cross-resistance strength value of 0.3 (left) or a cross-resistance value of 0.5 (right). In each of these panels, the combination drug dose is fixed at 0.4 per drug and the sequential drug doses are fixed at 0.8 for each drug. C-D) Average frequencies of mutants resistant to the first drug. E-F) Average frequencies of mutants resistant to the second strike. G-H) Average frequencies of cross-resistant mutants. Comparing each pair of panels, the increase in cross-resistance strength has little effect on the frequencies of mutants resistant to only the first or second strike. Conversely, cross-resistant mutants increase in frequency much more rapidly when cross-resistance strength increases, especially in the case of combination therapy. Note that the *y*-axis scales on panels G and H differ by roughly an order of magnitude.

When cross-resistance mutations are present and sufficiently strong, and the mutation rate is sufficiently high, optimally timed sequential therapy outperforms combination therapy (Figs 5B and Fig D in S1 Text). In the case of the combination dose being half the sequential dose (thereby ensuring the same total dose regardless of protocol), sequential therapy outperforms combination therapy only when the strength of cross-resistance is higher than 0.4 (i.e., a cross-resistant mutation in the presence of a single drug provides a fitness benefit greater than 40% of that provided by the drug-specific resistance mutation). For example, the extinction probability is 0.1 for combination therapy and 0.63 for optimally timed sequential therapy when the combination dose is 0.4, the sequential dose is 0.8, and the strength of cross-resistance is 0.5 (Fig 5B). As cross-resistance strengthens, the extinction probably decreases dramatically for both treatment strategies, although it remains higher for optimally timed sequential therapy.

The reason for the better performance of sequential therapy in the presence of strong cross-resistance can be seen by comparing the dynamics of resistant mutants during treatment between a weak cross-resistance scenario and a strong cross-resistance scenario. For mutants resistant to the first and second drug, the patterns of allele frequency change do not drastically differ for either sequential or combination treatment when comparing a cross-resistance of 0.3 to a cross-resistance of 0.5 (Fig 5C, 5D, 5E, 5F). The dynamics of cross-resistant mutants during combination therapy, however, do change when cross-resistance increases from 0.3 to 0.5 (compare Fig 5G, 5H, noting the different *y*-axis scales): cross-resistance mutations increase in frequency much faster when the strength of cross-resistance is higher. In contrast, during sequential treatment, the effect of the first strike on cross-resistant mutants occurs in two phases: in the first phase, cross-resistant mutants rise in frequency proportionately with the strength of cross-resistance, because they have higher fitness than drug-sensitive mutations. Mutations conferring resistance to drug 1 alone also increase in frequency during this phase, albeit to a much greater extent (Fig 5D, 5E). In the second phase, once fully drug-sensitive cells have been largely eradicated by the treatment, the application of the first drug results in selection against cross-resistant mutants because the selective advantage of mutations that confer resistance specifically to drug 1 is greater than that of cross-resistant mutations (Fig 5G, 5H). This effect results in a decline in the frequency of cross-resistant cells that contrasts with the increase in frequency of those mutants for the duration of treatment in combination therapy.

The dynamics of cross-resistant mutants explains two phenomena. The first is the precipitous drop in efficacy for both combination and sequential therapy at high values of cross-resistance due to cross-resistance mutations rising to higher frequencies during both therapies. The second phenomenon is the higher efficacy of sequential therapy compared to combination therapy once cross-resistance is sufficiently strong. Strongly cross-resistant mutations have the potential to rescue the cancer cell population in both combination and sequential therapy, but optimally timed sequential therapy can sufficiently decrease the frequency of these cross-resistant mutants (after an initial increase) to effectively limit preexisting genetic resistance to the second strike.

### Equal drug doses in combination therapy are optimal regardless of resistance mutation rates

Given the importance of the mutation rate to combination therapy efficacy, we tested whether there is an optimal ratio of drug doses during combination therapy if the two resistant mutations have different mutation rates. To achieve this, we carried out simulations involving only combination therapy, fixing the mutation rate for resistance to the second drug and varying the mutation rate of resistance to the first drug. As outlined in the Methods, we varied the drug dose ratio but kept their combined effect constant so that the likelihood of death for treatment-sensitive cells was the same across all simulations. Our results (Fig E in S1 Text) indicate that regardless of the difference in drug mutation rates, the highest chance of inducing population extinction is achieved when the doses of the two drugs in the combination is 1:1. This makes sense as this scenario poses the greatest evolutionary challenge for the population in that both treatments need to be overcome to likely have significant rebound. Thus, our evolutionary rescue model suggests that it may be preferable to use a combination of treatments where each drug has a similar efficacy at killing cancer cells.

### Extinction therapy dynamics appear largely similar if treatment resistance is a quantitative trait

So far, our simulations have treated resistance as a discrete trait. Evolutionary rescue has also been modelled as a quantitative trait [36] where resistance involves multiple loci of modest effect. We tested whether the results above hold in scenarios where resistance is conferred by the cumulative effect of multiple mutations. The contribution of each mutation to resistance was drawn from a lognormal distribution as described in the Methods. We find that an optimal lag time still exists in these simulations, and that it remains relatively independent of the mutation rate for resistance (Panel A in Fig F in S1 Text). However, the peaks of extinction probability around the optimal lag times are not as sharp as those in discrete resistance simulations, and when the mutation rate is high the extinction probability is considerably lower than for the discrete model (compare to Fig 3).

We also examined the impact of varying drug doses under this quantitative trait model and draw similar conclusions as under our discrete trait model: optimal lag times are relatively independent of mutation rates regardless of which drug has a lower dose, optimal lag times remain unchanged when drug 2 has the smaller dose, and optimal lag times are higher and extinction probabilities are lower when drug 1 has the smaller dose (Panels B and C in Fig F in S1 Text).

## Discussion

### Evolutionary rescue models reveal when the efficacy of combination therapy is greater than sequential therapy

We performed forward-in-time agent-based population genetic simulations to evaluate the effectiveness of several different treatment strategies under an evolutionary rescue model. Under a sequential two-strike “extinction therapy” regime, we showed that the timing of the switch from the first drug to the second has a marked effect on the probability of the cell population going extinct (Fig 3). We also found that using the more effective drug first and the less effective drug second leads to substantially higher extinction probabilities than vice-versa (Fig 4). Furthermore, our simulations show that combination therapy forms a formidable barrier to the evolution of resistance when no mutations conferring cross-resistance exist, outperforming even optimally timed sequential therapy involving much stronger drugs than those used in the combination therapy (Fig 5A). This implies that combination therapies with lower doses may be preferable to sequential therapy using drugs that achieve a higher kill rate, because the probability of adapting to *each* drug in combination is low so long as resistance mechanisms are independent—this result is consistent with insights gained from studies of pest control and antimicrobial treatment strategies [66,67]. We note that these results are in line with another potential benefit of deploying combinations of drugs that attack different targets in a tumor cell: the time and cost of developing and evaluating new treatments could be significantly reduced by constructing a cocktail of existing pharmaceuticals rather than designing new drugs [42]. We again stress that the two “drugs” in our sequential therapy model could each themselves represent drug combinations, and although it may be desirable to further reduce the probability of evolutionary rescue by further combining these two strikes into a single combination, in some cases toxicity concerns may preclude this possibility.

On the other hand, even drug combinations that target distinct pathways of a cancer cell can often be overcome through a single mechanism that produces multidrug resistance such as elevated xenobiotic metabolism, increased growth factor production, etc. [68,69]. Our simulations show that when mutations that confer sufficient levels of cross-resistance can occur, combination therapy can yield worse outcomes than sequential therapy (Fig 5B). The superior effectiveness of two-strike extinction therapy when cross-resistance is moderately strong (e.g., 0.5, as in Fig 5H) is because, under combination therapy, cross-resistant mutations increase in frequency at a higher rate than when cross-resistance is weak (e.g., 0.3, as in Fig 5G) and confer a sufficiently strong fitness advantage to have an appreciable chance of causing evolutionary rescue. Optimally timed sequential therapy, on the other hand, results in selection against cross-resistant mutations during the latter phase of the first strike, and this holds either when cross-resistance is weak or moderate (Fig 5G,5H), thereby diminishing the reservoir of genetic variants conferring preexisting resistance to the second strike.

However, as the degree of resistance conferred by cross-resistance mutations approaches that of the single-drug resistance mutations, not only does combination therapy have very low efficacy, but sequential therapy will fail at a high rate because treatment with drug 1 no longer effectively selects against cross-resistant mutations; these cross-resistance mutations will be able to respond immediately to the new selective pressure posed by the second strike. We also show that with combination therapies it is optimal to use drug dosing where each drug kills cancer cells at the same rate, regardless of whether resistance to one drug arises at a higher rate than the other. Overall, our results support the need for developing drug combinations that are recalcitrant to the evolution of multidrug resistance [70], and suggest that when cross-resistance cannot be avoided, two-strike extinction therapy strategies may in some cases yield better outcomes.

### *In silico* trials reveal variation in outcomes and optimal timing of treatment change among synthetic patients

We conducted *in silico* patient trials to investigate the evolutionary dynamics of two-strike extinction therapy in greater detail. We found that for many patients the optimal time to switch from the first to the second drug was at or near *t*_*min*_, the time at which the cancer cell population reaches its minimum and begins to rebound. This result is intuitive because the lower the population size, the fewer cumulative opportunities it will have to adapt to the second strike via new mutations (unless resistance to the second drug happens to already be present in the population; discussed below). Yet, the window of opportunity to switch therapy following *t*_*min*_ may differ among patients. We do find that, if one switches treatments shortly after *t*_*min*_, the probability of extinction is often near its maximum (Fig 2). However, we note that because the absolute amount of growth occurring immediately after *t*_*min*_ is relatively minimal (Figs 1 and 2), determining *t*_*min*_ may not be feasible in practice until well after it has occurred. Thus, if one waits until observable progression to initiate the second strike, it may already be too late. We therefore argue that developing approaches to accurately predict *t*_*min*_ for a given patient based on a limited number of scans/assays may prove key toward improving patient outcomes.

Our *in silico* trials show how adaptation to a treatment removes genetic diversity that could confer resistance to a second treatment. It is for this reason that in those patients for whom mutations conferring resistance to drug 2 were present at *t*_*min*_, we observed that the probability of extinction was very low if the second strike began at *t*_*min*_, but much higher if the duration of the first strike was slightly extended in order to remove these drug 2 resistance mutations. This illustrates that the success of two-strike extinction therapy shares some similarity to the strategy of imposing an evolutionary “double bind,” wherein resistance to one treatment confers susceptibility to another [71,72]. In our case this is a epiphenomenon arising from the reduction in tumor heterogeneity caused by selection rather than a direct phenotypic cost of resistance. In other words, the benefit of the first treatment is not only to shrink the size of the cancer cell population, but also to reduce the amount of diversity that could allow the population to immediately adapt to the second strike [73] (discussed further below). If treatments are switched too early, mutations conferring resistance to the second strike may remain present in the cell population, while waiting too long allows the population to rebound and allows mutations conferring resistance to the second strike to randomly (re-)emerge in the population.

Our results also imply that the strategy of repeated changes to the selective environment (i.e., treating a patient with a series of drugs used one after another in sequence) can further increase the probability of extinction. This is consistent with previous theoretical studies that have showed that frequent or continual environmental change can drive even initially very robust and adaptable populations to extinction [74,75]. Relatedly, Patil et al. recently showed that two-strike extinction therapy is much less likely to succeed for very large cell populations [50]. In such cases, extending treatment by adding more drugs to the sequence may be required to consistently achieve extinction, similar in principle to the 3-stage approach that has proved so effective in treating pediatric cases of acute lymphoblastic leukemia [76]. It has also been noted that when environmental stochasticity is present in addition to a major environmental shift (i.e., treatment), extinction probability is further increased (e.g., [77]), a fact that future treatment strategies may be able to exploit.

### Pre-existing treatment resistance is likely to be the primary cause of evolutionary rescue in cancer

Our results imply a prominent role of standing variation in determining the probability of evolutionary rescue during sequential therapy. Indeed, the primary driver of the probability of extinction during our simulations appears to be the probability that the cancer cell population contains at least some degree of resistance to the second drug prior to the treatment switch (Fig 3D). As shown in Table A in S1 Text, when resistance to the second drug is already present in the population, evolutionary rescue almost always occurred in our simulations, and conversely extinction was nearly guaranteed when no such resistance was present at the time of the second strike. We note that Orr and Unckless [39] describe the conditions in which rescue is more likely to occur via standing variation than de novo mutations arising after the environmental shift, but those conditions were derived under the assumption that selection is much weaker than the drug dose effects we consider here. Nonetheless, our results suggest that, in two-strike cancer therapy, standing genetic variation may be the far more likely cause of rescue. Thus, successful treatment strategies should seek to not only kill as many cancer cells as possible, but also to remove standing genetic variation. While these goals are often in alignment, this is not always the case (i.e., when continuing treatment with drug 1 for a brief period after reaching *t*_*min*_ removes genetic diversity even though the cancer cell population is recovering).

We note that our conclusions regarding the optimal timing of the second strike are largely in agreement with those of a recent study by Patil et al. [50] who constructed an analytical model of evolutionary rescue under a similar two-strike extinction therapy scenario as that examined here. Specifically, Patil et al. also found that the nadir of the population after the first strike is usually the optimal point to switch therapies, and that striking slightly after the nadir still yields good results. However, Patil et al. found that including a cost of resistance induces a more complex relationship where, if the second strike begins before the nadir, the extinction probability remains high for a longer duration when the first drug’s dose is intermediate and the second drug’s dose is high. This is because the population decline during the first strike is slower if the drug dose is not too high, and this allows more time for selection against cells resistant to the second drug, as these cells are experiencing the cost of resistance but no selective benefit. This result underscores the potential for resistance costs to qualitatively affect evolutionary dynamics during extinction therapy. The impact of the presence and strength of costs of resistance should thus be considered in future studies of not only the timing of two-strike extinction therapy but its relative efficacy vis-à-vis combination therapy.

### A quantitative trait model of resistance behaves similar to the discrete-trait model

Finally, the genetic architecture of resistance appears to have only modest implications for the effectiveness of sequential therapy. We observed similar dynamics whether treatment resistance is a discrete trait or a quantitative trait where the degree of resistance is the sum of the effects of multiple mutations. However, we note that in the latter scenario extinction probabilities display broader plateaus around the optimal lag times (Panel A in Fig E in S1 Text). This may suggest that, all else being equal, sequential therapy involving treatments that exert highly polygenic selection on cancer populations may require less precision in the timing of the second strike, whereas treatments for which resistance is governed by a single locus may in some cases yield poor outcomes if the timing of the second strike misses the optimum by a relatively small period of time (Fig 2).

### Agent-based simulations are highly flexible and can readily be extended to other scenarios of evolutionary rescue

This study adds to the growing body of work showing that modeling the dynamics of evolutionary rescue can inform optimal treatment strategies and timings. Here, we showed that a relatively simple agent-based population genetic simulation model of evolutionary rescue can reveal key dynamics underlying the emergence of resistance to cancer treatment. Moreover, the simulation approach that we adopted here is flexible, and can easily adapted to model additional treatment strategies (e.g., adaptive therapy; [54,78,79]) and to incorporate additional complexities into our model of cancer cell population evolution, adaptation, and extinction. Such features could include but are not limited to spatial heterogeneity and dynamics [80–82], Allee effects [83–86], treatment refugia [87], non-genetic resistance mechanisms such as epigenetic responses [88] and transitions to drug-tolerant cell states [89–91], fitness costs of resistance [92], and increasing rates of resistance mutations over time resulting from increased genomic instability [93]. More broadly, our study demonstrates the potential of flexible forward population genetic simulations of evolutionary rescue scenarios for investigating the dynamics of diverse treatment/management strategies for cancer and other diseases/organisms where the emergence of resistance is a common occurrence (e.g., antibiotic resistance, pesticide resistance, etc).

## Supporting information

S1 Text Supplementary tables and figures. Contains Figs A - F and Table A as referenced in this manuscript.
(DOCX)
