# Supplementary material for: Evolutionary rescue model informs strategies for driving cancer cell populations to extinction
Source: PLoS Comput Biol. 2026 Sep 18;22(9):e1014803. doi: 10.1371/journal.pcbi.1014803 (PMC13722315; doi:10.1371/journal.pcbi.1014803)
Supplement: Supplementary file 1 [file pcbi.1014803.s001.docx]

**Fig A. Conceptual simulation overview.** The cancer cell population expands stochastically from a single non-resistance cell until reaching a population of within 10% of $2^{20}$ cells. The first strike is then applied continuously for a specific number of cycles (the lag time) before the second strike is then applied continuously until the population reaches extinction or rescue. Cells can accumulate mutations that confer resistance to each drug at any point during the simulation.

**Fig B. Using a lower-dose drug first reduces overall treatment success and shifts the optimal lag time higher.** A) The average frequency of mutants resistant to the second strike ($f$) just before switching treatment at various lag times and mutation rates when the dose of the first drug is lower (same simulations as shown in Fig 4A). Black stars represent the optimal lag time(s). The average frequency is not strongly related to mutation rate, lag time, or extinction probability. B) The average value of $f$ just before switching treatment at various lag times and mutation rates when the dose of the second drug is lower (same simulations as in Fig 4B). The average **value of** $f$ is again not strongly related to mutation rate, lag time, and extinction probability. C) The average **value of** $f$ right before the optimal lag for each mutation rates. Blue line indicates simulations where the second drug has a higher dose and orange line indicates simulations where the first drug has a higher dose. While the mean frequency in the former case is sometimes higher, the mean values of $f$ for these two strategies do not diverge as the mutation rate increases, even though the extinction probabilities diverge as mutation rate increases (see Fig 4A,B). D) The median value of *t_min_* for both the best-drug-first and best-drug-second cases. The population reaches this minimum faster when the first drug dose is higher. E) Minimum population size during the first strike for both cases. The population declines to a lower median value across all mutation rates when the first drug dose is higher.

**Fig C. Comparison of combination therapy and sequential two-strike therapy protocols across various mutation rates and drug combination doses in the absence of cross-resistance mutations.** Black text indicates the fraction of extinct replicates for combination therapy and at the optimal second-strike lag for sequential therapy for each drug combination dose. The dose of both drugs in the sequential therapy scenarios is fixed at 0.90. Each panel represents a different mutation rate, with panel (A) representing a mutation rate of ${2\times10}^{-5}$, increasing by ${2\times10}^{-5}$for each panel until reaching ${2 \times10}^{-4}$ in panel (J). Combination therapy outperforms optimally timed sequential therapy across all parameter combinations.

**Fig D. Comparison of combination therapy and sequential two-strike therapy protocols resistance in the presence of cross-resistance mutations across various mutation rates and strengths of cross-resistance.** Black text indicates the fraction of extinct replicates for combination therapy and at the optimal second-strike lag for sequential therapy for each strength of cross-resistance. The dose of both drugs in the combination protocol is fixed at 0.4, while the dose for both drugs in the sequential therapy is fixed at 0.8. Each panel represents a different mutation rate, with panel (A) representing a mutation rate of ${2 \times10}^{-5}$, increasing by ${2 \times10}^{-5}$for each panel until reaching ${2\times10}^{-4}$ in panel (J). With the introduction of cross-resistance, sequential therapy outperforms combination therapy at the doses chosen, even at low levels of cross-resistance.

**Fig E. Fraction of extinct replicates at various doses of the first drug and rate of mutation of the second drug in a combination therapy scenario.** The mutation rate for the second drug is fixed at ${2\times10}^{-5}$, and the dose for the first drug is always chosen so that the probability of survival for non-resistant cells is the same. An equal or close-to-equal dose of both drugs is the optimal choice regardless of the mutation rate of resistance to the second drug. Black stars are drawn for each row at the combination dose with the highest extinct fraction, with multiple stars per row in case of a tie.

**Fig F. Results of simulations where resistance is considered a quantitative trait rather than a binary one, with 10 mutations of one type on average conferring full resistance in a cell to the corresponding drug.** (A) The effect of the mutation rate and the second strike lag on the fraction of extinct replicates (extinct fraction), with the dose of both drugs fixed at 0.9. (B) The effect of the mutation rate and the second strike lag on the extinct fraction when drug 1 has a dose of 0.75 and drug 2 has a dose of 0.9. (C) The effect of the mutation rate and the second strike lag on the extinct fraction when drug 1 has a dose of 0.9 and drug 2 has a dose of 0.75. (E) The effect of the dose of both drugs and the second strike lag on the extinct fraction when the mutation rate is fixed at $6.4\times10^(-6)$. In general, conclusions from the corresponding binary trait resistance simulations hold, but the peaks of extinction probability around the optimal lag times are broader in the case of resistance as a quantitative trait.

Table A: Extinction probability vs. the frequency of mutations providing resistance to the second drug just before a second strike performed at *t_opt_*. This frequency is denoted *f_to_*. The number of replicates in each *f_to_* bin is also shown. This table was computed from the same simulation results summarized in Figure 3.

| Frequency of m2 mutations | Extinction probability  (95% C.I.) | Number of replicates in bin |
| --- | --- | --- |
| *f_to_* = 0 | 0.9904  (0.9832 – 0.9945) | 1244 (82.66%) |
| 0 < *f_to_* <= 1e-4 | 0.0000  (0.0000 – 0.6576) | 2 (0.13%) |
| 1e-4 < *f_to_* <= 1e-3 | 0.0643  (0.0342 – 0.1177) | 140 (9.3%) |
| 1e-3 < *f_to_* <= 1e-2 | 0.0000  (0.0000 – 0.0377) | 98 (6.51%) |
| 1e-2 < *f_to_* <= 1e-1 | 0.0000  (0.0000 – 0.1611) | 20 (1.33%) |
| 1e-1 < *f_to_* <= 1.0 | 0.0000  (0.0000 – 0.7935) | 1 (0.07%) |
